# Urban Birds as Antimicrobial Resistance Sentinels: White Storks Showed Higher Multidrug-Resistant *Escherichia coli* Levels Than Seagulls in Central Spain

**DOI:** 10.3390/ani12192714

**Published:** 2022-10-09

**Authors:** Bárbara Martín-Maldonado, Pablo Rodríguez-Alcázar, Aitor Fernández-Novo, Fernando González, Natalia Pastor, Irene López, Laura Suárez, Virginia Moraleda, Alicia Aranaz

**Affiliations:** 1 Deparment of Veterinary Medicine, School Biomedical and Health Sciences, Universidad Europea de Madrid, 28670 Villaviciosa de Odón, Spain; 2 Wildlife Hospital, Grupo de Rehabilitación de la Fauna Autóctona y su Hábitat (GREFA), 28220 Majadahonda, Spain; 3 Grupo de Estudio de la Medicina y Conservación de la Fauna Silvestre (GEMAS), 28220 Majadahonda, Spain; 4 Department Animal Health, Faculty of Veterinary Medicine, Universidad Complutense de Madrid, 28040 Madrid, Spain

**Keywords:** antimicrobial resistance, surveillance, urban wildlife, migratory birds

## Abstract

**Simple Summary:**

Antimicrobial resistance (AMR) has become a major health challenge of the 21st century. Several studies confirm the potential role of wildlife as sentinel for pathogens surveillance. Moreover, the presence of AMR bacteria in the wildlife can be considered as a good indicator of anthropization level on the ecosystem. The fast increase in AMR worldwide has been enhanced by several factors as globalization and migration. The study of antimicrobial resistance in wild birds is of great importance, as they can travel hundreds of kilometers and disseminate pathogens and AMR across different regions or even continents. The aim of this study was to compare the level of AMR in three bird species: white stork (*Ciconia ciconia*), lesser black-backed gull (*Larus fuscus*) and black-headed gull (*Chroicocephalus ridibundus*). For the analysis, 17 antibiotics from the most representative classes were tested by disk-diffusion method. Results showed 63.2% of seagulls and 31.6% of white storks as carriers of antimicrobial-resistant *Escherichia coli*, and from all of them, 38.9% were considered multi-drug resistant. Betalactamics, quinolones and tetracyclines were the antibiotic classes with the highest rate of AMR.

**Abstract:**

The presence of AMR bacteria in the human–animal–environmental interface is a clear example of the One Health medicine. Several studies evidence the presence of resistant bacteria in wildlife, which can be used as a good indicator of anthropization level on the ecosystem. The fast increase in AMR in the environment in the last decade has been led by several factors as globalization and migration. Migratory birds can travel hundreds of kilometers and disseminate pathogens and AMR through different regions or even continents. The aim of this study was to compare the level of AMR in three migratory bird species: *Ciconia ciconia*, *Larus fuscus* and *Chroicocephalus ridibundus*. For this purpose, commensal *Escherichia coli* has been considered a useful indicator for AMR studies. After *E. coli* isolation from individual cloacal swabs, antimicrobial susceptibility tests were performed by the disk-diffusion method, including 17 different antibiotics. A total of 63.2% of gulls had resistant strains, in contrast to 31.6% of white storks. Out of all the resistant strains, 38.9% were considered multi-drug resistant (50% of white storks and 30% of seagulls). The antibiotic classes with the highest rate of AMR were betalactamics, quinolones and tetracyclines, the most commonly used antibiotic in human and veterinary medicine in Spain.

## 1. Introduction

In the recent centuries, the anthropization of ecosystems has forced several animal species to change their biology and adapt to human presence, coexisting today as urban wildlife. Urban areas give them unlimited resources to live, feed and reproduce [1]. Currently, white storks (*Ciconia ciconia*) are considered migratory birds turned into sedentary wildlife. Similarly, lesser black-backed gulls (*Larus fuscus*) and black-headed gulls (*Chroicocephalus ridibundus*) traditionally settle on coastal areas, moving short distances to warmer latitudes in winter. The constant availability of food and other resources in cities has favored the establishment of some populations of the three species as residents, shortening or stopping migration [2,3].

In this sense, landfills constitute an important food source for opportunistic wildlife. However, the nutritional score of this food is poor and incomplete and involve several risks for animal health as the accumulation of contaminants, such as heavy metals, pesticides or drugs (e.g., anti-inflammatories and antimicrobials) [2,4]. The accumulation of organic waste in landfills, plus the inappropriate management of antibiotic residues, have favored the development of antimicrobial resistance (AMR) and the potential acquisition of resistant bacteria by animals who feed at these points [5,6]. One of the key facts on AMR dissemination is the horizontal transmission of antimicrobial resistance genes (ARGs) between bacteria, no matter if they are the same bacterial species or not. Thus, commensal and environmental bacteria can acquire these ARGs and serve as amplifiers that perpetuate the persistence of genes in individuals or even ecosystems [7,8]. Moreover, AMR should be considered a zoonosis as ARGs run between the human–animal–environment interface easily [9]. Now, AMR represents one of the biggest challenges in medicine, as it has been linked to nearly 5 million deaths worldwide in 2019 [10,11]. *Escherichia coli* is a commensal bacterium in the gastrointestinal tract of a wide range of hosts that has a high survival rate on the environment, which make it a good indicator for AMR studies [12]. Instead of being mainly saprophytic, some serovars can cause disease, called colibacillosis, which is considered relevant to public and animal health as it is the fourth most reported foodborne gastrointestinal infection in humans in the European Union (EU) [13]. Moreover, *E. coli* has been described as one of the most frequent resistant bacteria in human and animal medicine, which make the treatment of colibacillosis more difficult [14].

However, AMR is not only a human health issue. Several studies have confirmed the presence of AMR in livestock, companion animals and wildlife [6,12,14,15,16]. The prevalence of AMR largely depends on animal species and regions, but resistant bacteria have been detected even in Antarctica [17,18]. The presence of AMR in wildlife is directly related to the pressure of human activity on the ecosystems. Therefore, some wildlife species can be considered sentinels for AMR pressure in the environment, and AMR surveillance in wildlife should be a priority [9,19]. Among bird species, seagulls are considered good sentinels for AMR studies, and high rates of resistant *E. coli* have been reported in those species [20,21,22,23,24]. There is limited information regarding storks, but recent studies have also described the presence of antimicrobial resistance in *E. coli* in white storks [5,25,26]. 

In this context, the aim of this study was to compare the presence of AMR *E. coli* in white storks and two species of seagulls in central Spain, more than 400 km (≈250 miles) inland. 

## 2. Materials and Methods

### 2.1. Study Population and Sample Collection

From October 2018 to May 2019, all the white storks (*Ciconia ciconia*), lesser black-backed gulls *(Larus fuscus*) and black-headed gulls (*Chroicocephalus ridibundus*) admitted at the Wildlife Rescue Center (WRC) managed by Grupo de Rehabilitación de la Fauna Autóctona y su Hábitat (GREFA) were examined and sampled. Handling procedures complied to European (Directive 2010/63/EU) and Spanish legislation (Royal Decree 53/2013) [27,28]. 

This GREFA WRC is located in central Spain (Madrid) and admits almost 7000 wild animals per year, including all kind of birds, mammals, and reptiles of native Iberian fauna. GREFA’s aim is to recover and release them back into the wild. The main reasons of admission to the WRC are related to human activities: hunting, accidents with power lines (electrocution or traumas), windows or cars, among others. Additionally, natural diseases of wildlife are another cause of admission at the WRC. In fact, WRCs can be considered as passive health monitoring centers for wildlife. 

**Figure 1:**
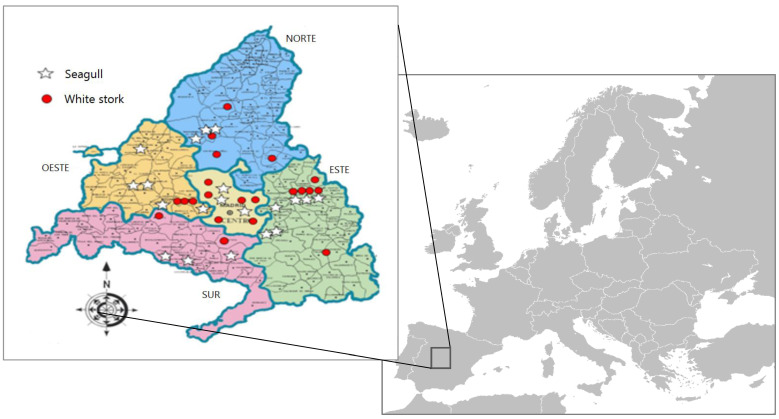
Origin of the animals included in the study.

During the first examination, a cloacal swab was taken from each animal for *E. coli* isolation and AMR detection, prior to any treatment. The samples were conserved on a Cary Blair transport medium (Deltalab, Barcelona, Spain) at 4 °C and processed within 24 h from the collection. Information about species, age, area of origin, proximity to landfills and clinical data from each animal was recorded when possible. Moreover, historical information about ringed birds was facilitated by official organisms from the countries where they had been ringed. Animals were classified by age in three groups, based on the feather development and phenotypic changes: nestling (including in this group fledglings), young and adult. According to the origin, five different regions were established to assess potential geographical differences: center, north, south, east, and west of the Community of Madrid (Figure 1).

### 2.2. Microbiological Analysis

Samples were plated onto MacConkey agar (Oxoid Ltd., Basingstoke, United Kingdom) and incubated at 37 ± 1 °C for 24 h. Next, a single colony morphologically compatible with *E. coli* was subcultured on a Columbia agar plate (Oxoid Ltd., Basingstoke, UK) and incubated again at 37 ± 1 °C for 24 h in order to get a monoclonal culture, which was collected and stored at −20 °C for further analyses. Bacterial identification was confirmed by Gram stain and classical biochemical tests, including catalase, potassium hydroxide (KOH), oxidase, glucose fermentation and motility tests [29]. Additionally, seven random isolates were tested using API (Analytical Profile Index) 20E strips (BioMérieux, Marcy l’Etoile, France). All the confirmed strains were stored in cryovial with nutritive broth and glycerol (80%: 20%, respectively) at −80 °C for further analysis. 

Antimicrobial susceptibility test was performed according to the disk diffusion (Kirby–Bauer) method and the European Committee on Antimicrobial Susceptibility Testing (EUCAST) guidelines [30]. Isolates were recovered from cryovials and spread on Columbia Agar with 5% sheep blood (Becton Dickinson GmbH, Heidelberg, Germany). The inoculum suspension was prepared in sterile 0.8% saline solution to a turbidity 0.5 McFarland. Then, the inoculum was transferred onto Mueller–Hinton agar (Becton, Dickinson Gmb, Heidelberg, Germany) and antimicrobial disks were added to the surface. A total of 17 antimicrobials from nine classes were tested, most of them recommended for indicator commensal *E. coli* (Decision EU 2020/1729) (Table 1) [31]. After 18–20 h at 36 ± 1 °C, sensitivity or resistance was determined by growth inhibition diameter regarding standardized EUCAST breakpoint tables [32], except for ceftiofur, enrofloxacin and tetracycline that were evaluated according to Markey et al. [29]. Multidrug resistance (MDR) was considered when the isolate was non-susceptible to at least one antimicrobial agent in three or more different classes of antimicrobial [33].

### 2.3. Statistical Analysis

Statistical analysis was done using a commercially available software application (SPSS 21.0 software package: SPSS Inc., Chicago, IL, USA, 2002). For the presentation of mean results, 95% confidence values were calculated. Different statistical tests were performed to assess the relationship between the presence of antimicrobial resistances and different variables (species, age, origin and pathology). Chi-square and Fisher’s exact test were employed to study parametric variables, and Mann–Whitney U test for non-parametric variables. A two-tailed *p*-value ≤ 0.05 was considered to indicate a statistically significant difference.

## 3. Results

A total of 40 animals were included in the study: 20 white storks, 16 lesser black-backed gulls and 4 black-headed gulls. Because of the small number of black-headed gulls included on the study, they have been grouped with lesser black-backed gulls for the statistical analysis (seagulls from now on). Animals were admitted at GREFA WRC because of different conditions, mainly trauma for white storks and botulism or trauma for seagulls. Classification by age showed that 20% were nestlings (8/40), 20% young (8/40) and 60% adults (24/40).

Individuals included in the study were firstly found and collected in areas with human activity, where some resident populations have been established and feed regularly in landfills. However, some animals maintain recent migratory habits, as detected by leg rings. Regarding their geographical origin, 20% were from the Center of the Community of Madrid (8/40), 10% from the south (4/40), 20% from the west (8/40), 20% from the north (8/40) and 30% from the east (12/40). 

*E. coli* was isolated from 38 animals: 19 storks and 19 seagulls. Overall, 47.4% (45.9–48.9%) of *E. coli* isolates were resistant to at least one of the 17 antimicrobials tested (18/38). The higher percentage of resistance was found in (in decreasing order): ampicillin (13/18), ticarcillin (13/18), nalidixic acid (9/18), tetracycline (8/18) and enrofloxacin (7/18). The resistance to ampicillin was always related to resistance to ticarcillin. Only one isolate was resistant to aztreonam. All isolates were susceptible to cefoxitin, ceftiofur, cefotaxime, imipenem and amikacin.

The percentage of AMR observed for each antimicrobial and the phenotypic profiles are detailed in Table 2. 

**Figure 2:**
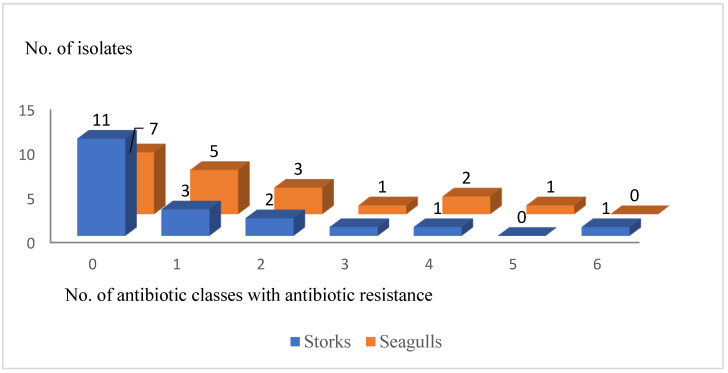
Number of *Escherichia coli* isolates showing sensitivity (0) or phenotypic resistance to one or more antibiotic classes (one to six) according to bird species.

From all the resistant *E. coli* isolates, 38.9% (35.16–42.64%) were considered MDR (7/18). The distribution of non-susceptible isolates to one or several antimicrobial classes is detailed in Figure 2.

Regarding bird species, 31.6% (28.8–34.4%) of *E. coli* isolates from the white storks (6/19) were resistant to at least to one antimicrobial, and three of these isolates were considered MDR. The six isolates were resistant to penicillins and one also showed resistance to aztreonam. Additional resistances were found in four isolates. Two MDR isolates showed resistance to four and six antimicrobial classes, combining (in addition to penicillins) quinolones, tetracycline, trimethoprim-sulfamethoxazole and gentamicin or chloramphenicol (Table 2).

Among seagulls, 63.2% (60.5–65.8%) of *E. coli* were resistant (12/19), and four of them were considered MDR. The *E. coli* isolated from two of the four black-headed gulls showed resistance: one to tetracyclines and the other one had an MDR pattern (AMP-TIC-CAZ-TET). Patterns showed more diversity than those observed in white storks. Resistance to penicillins was found in seven isolates, resistance to quinolones in seven isolates and to tetracycline in six isolates. The four MDR isolates showed resistance against three, four or five categories, combining penicillins and/or quinolones, with trimethoprim-sulfamethoxazole, tetracycline, chloramphenicol or gentamicin (Table 2).

No significant differences were found between the presence of AMR and the species (*p* = 0.11), age (*p* = 0.59), origin (*p* = 0.47) and pathology (*p *= 0.24); neither between the existence of MDR and the same variables (*p* = 0.78, *p* = 0.15, *p* = 0.11 and *p* = 0.46, respectively).

## 4. Discussion

This study shows a high proportion of AMR *Escherichia coli* isolates (47.4%) from white storks, lesser black-backed gulls, and black-headed gulls, three urban bird species that have modified their feeding habits and migratory behavior in recent years. Despite the fact AMR should be expected in a lower proportion in wild birds, urban birds have a closer contact with human garbage, increasing the risk of acquiring AMR [24,34,35]. Free-living wildlife is never supposed to have received antibiotic treatment; thus, the main source of this resistance may be the interaction with human wastes and sewage. Several publications highlight the development of AMR in bacteria present in landfills and water treatment plants [36,37]. The trend of white storks, black-backed gulls and black-headed gulls wintering in Spain to feed on landfills observed in recent years would fit the hypotheses of this exposure [3,38]. 

It is known that antibiotic consumption promotes the development of AMR in animals [39]. In the EU, the most employed antibiotics in human medicine are betalactamics, macrolides and quinolones, while in veterinary it is betalactamics and tetracyclines [40]. Betalactamics and quinolones were shown to be the antimicrobial classes with the highest rate of resistance in the present study. According to the Spanish Agency for Drugs and Medical Devices (AEMPS), the antibiotic with the highest percentage of AMR in *E. coli* in Spain in 2017 was ampicillin in humans and livestock, with rates of 65% and 72.81%, respectively [39]. The extended use of this antibiotic agrees with the high percentage of ampicillin-resistant strains detected in both white storks and seagulls.

The prevalence of AMR found in the seagulls included in the present study (63.2%) is higher compared to those published in EU countries. Among the antimicrobials tested by other authors in samples from seagulls from the Mediterranean countries, the present study showed similar results: antimicrobial classes with higher resistance were penicillins, tetracyclines and quinolones [9,15,20,22,41,42]. Previously, Stedt et al. assessed the AMR presence in seagulls from the Spanish Mediterranean coast among other regions and the presence of AMR is similar between both studies (61.2% Stedt vs. 63.2%) [22]. It is interesting to note that, despite the high level of AMR, all the isolates were susceptible to cephalosporins (except one to ceftazidime) and carbapenems. Resistance to these antimicrobials has been raised in seagulls worldwide [9,43,44,45] and has also been described in the Iberian Peninsula [21,46,47]. It is tempting to speculate if resistances to cephalosporins found in coastline birds are related to seawater contaminated with human sewage, and thus not affecting inland wild birds.

Regarding white storks, there is less information about the burden of AMR in *E. coli* in this species. Our results agree to those reported by Skarzynska et al. [48]: a high proportion of resistance to penicillins, quinolones and trimethoprim-sulfamethoxazole. However, Camacho et al. evaluated the AMR in white storks from central south Spain and found a higher level for gentamicin (44.8–46.7%) and enrofloxacin (40.2–41.4%), compared to our results (10.5%, both) [5]. Additionally, cefotaxime-resistant strains were detected in a high proportion (22–37.9%) while all our isolates were sensitive. A recent study that has analyzed the AMR on *E. coli* isolated from storks, including 12 antimicrobials [26], found a higher proportion of resistant isolates, compared to our panel, specifically to ampicillin (100%), nalidixic acid and ciprofloxacin (80%), tetracycline (67%) and gentamicin (33%) [26]. 

In addition, wild birds may harbor other relevant bacterial species, such as *Salmonella* and *Staphylococcus aureus*. *Salmonella* spp. isolates from white storks feeding on the same area of our study showed resistance to quinolones and ampicillin, which is in accordance with our results, but none was resistant to gentamicin and chloramphenicol [6]. Additionally, methicillin-resistant *S. aureus* has been isolated in tracheal samples from white storks (3.3%) [49]. In these studies, AMR carriage has been attributed to human residues exposure [5,6,26,49].

The rate of MDR in white storks (50%) is higher than that obtained in previous studies [5]. However, the proportion of MDR observed in seagulls (25%) agrees with those published by Stedt et al. (28.6–45.3%) [22]. 

Some species of wildlife had been suggested as sentinels for AMR surveillance in the ecosystems. However, sampling wildlife requires considerable logistical efforts and/or the handling of animals without interfering in their welfare. In this context, WRCs can be valuable resources [5]. One advantage is the success of the microbiological analysis, as the time between the sample collection and the arrival at the laboratory is shortened. *E. coli* was recovered from a higher proportion of sampled animals than in other studies [5,23,50].

Finally, assigning the source and dissemination of AMR is not easy and the role of wildlife as reservoirs of AMR for humans would require extended studies [19,50,51]. Two issues should be highlighted regarding the wildlife species studied here. First, some of these birds are migratory. In fact, two white storks and two lesser black-backed gulls sampled in this study had a leg ring from Belgium (stork no. 28) and Germany (stork no. 12, seagulls no. 11 and no. 16), respectively. Interestingly, stork no. 12 and seagull no. 11 carried MDR (penicillins, quinolones, tetracyclines, sulfonamides) and AMR-resistant *E. coli* isolates, respectively. It has been described that migratory birds can carry more AMRs than non-migratory [52], as the migratory patterns of wild birds can exponentially magnify the dissemination and acquisition of AMRs, even in remote regions [53]. Second, most of the animals sampled in this study had been brought into the WRC by citizens who found the animals, which implies a direct contact in the human–wildlife interface. Although the spillover of enteric pathogens from wild birds to humans is controversial [54], a close contact by handling an injured animal could represent a transference route in both directions for citizens and WRC staff [51].

Once a health issue is introduced into a wild population, its control is difficult to achieve. Thus, a key priority would be a prudent management of anthropogenic wastes and sewage to avoid the access of wild animals and prevent the transmission of AMR to wildlife.

## 5. Conclusions

In conclusion, our study describes the profiles of phenotypic AMR detected in *E. coli* isolated from white storks and seagulls in central Spain. These bird species can be considered suitable sentinels for AMR and MDR surveillance. The proportion of AMR and MDR detected in the present study is higher than the rates published by other authors, which could be in concordance with the upward trend of AMRs detection worldwide. In this context, an adequate management of antibiotic residues and urban waste should be a priority to prevent further AMR dissemination into wildlife.

## Figures and Tables

**Table 1:** Antimicrobial disks used in this study.

Class of Antimicrobial	Antimicrobial	Code	Concentration (µg)	Supplier
Penicillin	Ampicillin	AMP10	10	Bio-Rad^®^
	Ticarcillin	TIC75	75	Bio-Rad^®^
	Amoxicillin-clavulanic acid	AMC30	20–10	Bio-Rad^®^
Cephalosporine	Cefotaxime	CTX30	30	Bio-Rad^®^
	Cefoxitin	FOX30	30	Bio-Rad^®^
	Ceftazidime	CAZ30	30	Bio-Rad^®^
	Ceftiofur	XNL30	30	Becton, Dickinson^®^
Monobactam	Aztreonam	ATM30	30	Bio-Rad^®^
Carbapenem	Imipenem	IPM10	10	Bio-Rad^®^
Quinolone	Nalidixic acid	NAL30	30	Bio-Rad^®^
	Ciprofloxacin	CIP5	5	Bio-Rad^®^
	Enrofloxacin	ENR5	5	Oxoid^®^
Tetracycline	Tetracyclin	TET30	30	Bio-Rad^®^
Aminoglycoside	Amikacin	AKN30	30	Bio-Rad^®^, BD^®^
	Gentamicin	GNM10	10	Bio-Rad
Sulphonamide	Trimethoprim-sulfamethoxazole	SXT25	1.25–23.75	Bio-Rad^®^
Amphenicol	Chloramphenicol	CHL30	30	Bio-Rad^®^

Bio-Rad Laboratories^®^, Hercules, CA, USA; Becton, Dickinson GmbH^®^, Heidelberg, Germany; Oxoid Ltd., Basingstoke, United Kingdom.

**Table 2:** Phenotypic profiles of antimicrobial resistance in *Escherichia coli* by species and overall prevalence of resistant or intermediate results.

Species ^1^ Id. No.	Antimicrobials ^2^											
	AMP	TIC	AMC	CAZ	ATM	NAL	CIP	ENR	TET	GNM	SXT	CHL
Storks												
10												
12 *												
14												
22												
32												
36												
37												
40												
Seagulls												
1												
2												
3												
4 *												
8												
11 ^†^												
13 *												
18												
20												
21												
25												
29												
Prevalence ^3^ (%)												
R	34.2	34.2	10.5	-	2.6	21.1	15.8	18.4	21.1	10.5	13.2	7.9
I	-	-	-	5.3	-	2.6	2.6	5.3	-	-	-	-

Phenotypic resistance: dark grey, intermediate resistance: light grey. ^1^ Bird species: white storks (*Ciconia ciconia*), and seagulls sampled in the Community of Madrid (central Spain). Seagulls with * were black-headed gull (*Chroicocephalus ridibundus*), the rest were lesser black-backed gull (*Larus fuscus*). Individuals with ^†^ had a leg ring from an EU country. ^2^ Antimicrobials: AMP: ampicillin, TIC: ticarcillin, AMC: amoxicillin-clavulanic acid, CAZ: ceftazidime, ATM: aztreonam, NAL: nalidixic acid, CIP: ciprofloxacin, ENR: enrofloxacin, TET: tetracycline, GNM: gentamicin, SXT: trimethoprim-sulfamethoxazole, CHL: chloramphenicol. All isolates were sensitive to cefoxitin, cefotaxime, ceftiofur, imipenem and amikacin. ^3^ Prevalence according to 38 isolates (19 *E. coli*-culture positive animals each group). R: resistant strains, I: intermediate strains.

## Data Availability

The data that support the findings of this study are available on request from the corresponding author (A.A.).
